# The Sanitary Blocks of Hospitals

**Published:** 1920-04-10

**Authors:** 


### HOSPITAL CONSTRUCTION: THE NEW ERA.

### The Sanitary Blocks of Hospitals.

We published last week a short letter which Mr. Keith Young wrote as a rejoinder to the article in The Hospital of March 27 (p. 615). In his letter Mr. Young makes four points. His first is that a cross-ventilated lobby will not stop a strong draught working from the sanitary appliances towards the ward door. His second is that if the receptacle for bedpans waiting inspection is properly contrived no air can pass over or through it towards the wards.

Let us admit with regard to this point that a properly designed receptacle certainly should be a safeguard, and that a designer such as Mr. Young would probably make sure of it being innocuous, at least in intention, though he himself admits that in practice things get left open which should be closed. His third and fourth points are that we were wrong in excusing some of the features we see in American plans on the ground that (or in the hope that) they rely on mechanical ventilation, and, finally, that what we require in hospitals we should demand in our homes.

To begin at the end, we heartily agree that there are many hospital features which ought to find a place in domestic architecture. Perhaps Mr. Young also means that what is good enough for a well-designed house is good enough for a hospital. There is much truth in this also. But is it not a fact that we are all beginning to realise that even the simple watercloset of the ordinary house is the better for *some* degree of isolation? Good house designers certainly aim at this, and anyone who studies such matters during his visits to the homes of England cannot fail to realise how often a costly and otherwise well-designed house is marred by quite unnecessary "nastiness" in this particular.

We are not making the mistake that Mr. Young is opposed to a lobby between the sanitary places and the ward. His *bête noire* is not the existence of a lobby—which, indeed, is almost a necessity of the planning problem—but the *additional* "cut-off" lobby. Of course, if this is unnecessary Mr. Young is amply justified in condemning it as an unwarrantable expense. But the point is that in hospital construction we want not only to be sure, but to be doubly sure; and if it is reasonably certain that the "cut-off," or cross-ventilation, lobby deters air from passing direct from the regions of fæcal disposal to the sick-room, it seems at least an act of negligence to dispense with it. We venture to think that Mr. Young is wrong in asserting that a strong current of air from the sanitary block towards the wards has no chance of being deflected or deterred by the "cut-off." One has to remember that a draught is not being pushed, but being pulled.

So long as the sanitary group is more or less united as a single chamber or air vessel at one with the ward any formation of partial vacuum in the latter (such as is caused by a fireplace) must act directly as an attraction to the air in the former. And since a passage with open windows in each side is almost certain to be the subject of a cross-current—in at one side and out at the other—it stands to reason that the air in the remoter chamber (the sanitary group) is not only likely to be diverted if it enters the "cut-off" lobby, but is very unlikely to be stimulated towards the ward by any atmospheric conditions within the ward. We cannot but approve the courage of Mr. Young's position, but it is difficult to believe that it is not hazardous to a dangerous degree.

